# Intravenous Tranexamic Acid Improves the Intraoperative Visualization of Endoscopic Sinus Surgery for High-Grade Chronic Rhinosinusitis: A Randomized, Controlled, Double-Blinded Prospective Trial

**DOI:** 10.3389/fsurg.2021.771159

**Published:** 2021-11-16

**Authors:** Wenjing Yang, Haoling Gou, He Li, Ying Liu, Ying Wan, Chengshuo Wang, Guyan Wang, Luo Zhang

**Affiliations:** ^1^Department of Anesthesiology, Beijing Tongren Hospital, Capital Medical University, Beijing, China; ^2^Department of Anesthesiology, Beijing Renhe Hospital, Beijing, China; ^3^Department of Otolaryngology-Head and Neck Surgery, Beijing Tongren Hospital, Capital Medical University, Beijing, China

**Keywords:** tranexamic acid, hemostasis, chronic rhinosinusitis, endoscopic sinus surgery, Sonoclot analysis

## Abstract

**Objective:** Intraoperative bleeding during endoscopic sinus surgery (ESS) for high-grade rhinosinusitis can be serious and can further obscure the surgical field. This study was designed to evaluate the effect of tranexamic acid (TXA) on the surgical visualization of ESS for high-grade rhinosinusitis.

**Methods:** In total, 60 patients with high-grade chronic rhinosinusitis (Lund-Mackay score 12 or greater) treated by ESS were randomized into two groups: the control group (Group C) or the TXA group (Group T). Each group included 30 patients. Patients in Group T received intravenous TXA, and those in Group C received normal saline. The Boezaart grading scale (BS) score was assessed as the primary outcome. Total blood loss (TBL), whole blood coagulation, and fibrinolysis were assessed by Sonoclot analysis, and complications were recorded and compared between the groups.

**Result:** A significant difference was found in the BS score between Group T and Group C [2.02 (1.88–2.05) vs. 2.27 (2.13–2.41), *P* = 0.011]. Increases in platelet function (PF) and fibrin degradation time (FDT) were assessed during the operation and showed significant differences between Group T and Group C (*P* = 0.040 for PF; *P* = 0.010 for FDT). No difference in complications was found between the two groups.

**Conclusion:** A 15 mg/kg bolus of intravenous TXA before surgery can improve the surgical visualization of ESS for high-grade chronic rhinosinusitis without causing significant adverse effects. Intravenous TXA may be beneficial in ESS for high-grade chronic rhinosinusitis.

**Clinical Trial Registration:** https://www.chictr.org.cn/edit.aspx?pid=121653&htm=4.

## Introduction

Optimal visualization of anatomical structures and landmarks during endoscopic sinus surgery (ESS) is critical to minimize the risk of complications, such as injuries to the skull base, optic nerve, and internal carotid artery (1). Many techniques to control bleeding in other parts of the body cannot be applied in ESS because only one hand can be used to perform the work through a nostril, with the other hand holding the optic scope (2). In addition, intraoperative bleeding is especially serious in highly vascularized structures and can further obscure the surgical field. The Lund-Mackay score is considered to have a positive correlation with intraoperative bleeding and can be a predictive factor of intraoperative bleeding in ESS (3). Many efforts have been made to reduce intraoperative bleeding and improve the visualization of ESS, including topical decongestants, reverse Trendelenburg positioning, controlled hypotension, and total intravenous anesthesia, most of which have been demonstrated to be effective. However, for cases of serious inflammation with high-grade Lund-Mackay scores, a series of techniques should be applied to improve intraoperative visualization (3).

As an antifibrinolytic agent, tranexamic acid (TXA) has been demonstrated to prevent bleeding effectively in multiple clinical scenarios, including acute trauma, dental and obstetric procedures, orthopedic and cardiothoracic surgeries, hemoptysis, and epistaxis (4). A small number of studies have shown that the systemic administration of TXA can decrease intraoperative blood loss and operative time (5, 6). However, this finding is controversial (7), and adverse effects should be evaluated synchronously. Therefore, the current study aimed to evaluate the effect of systematic TXA on intraoperative visualization of ESS, blood loss, and surgical time, as well as the incidence of adverse effects. Coagulation and fibrosis profiles were also recorded and compared by Sonoclot analysis. We hypothesized that TXA would improve the intraoperative visualization of ESS for high-grade chronic rhinosinusitis. To the best of our knowledge, this is the first study investigating the effects of TXA on high-grade chronic rhinosinusitis.

## Patients and Methods

### Patients

This double-blinded, randomized controlled trial (RCT) was conducted at Beijing Tongren Hospital, reviewed and approved by the Ethics Committee of Beijing Tongren Hospital (TRECKY2020-065), and registered in the Chinese clinical trial registry (ChiCTR2100043139). Patients aged 18–65 years diagnosed with chronic rhinosinusitis and scheduled for elective ESS were examined by computed tomography (CT) 1–5 days before surgery. These cases were evaluated by the Lund-Mackay grading system, and those with a score of 12 or greater (out of a maximum of 24 points) (8), indicating an elevated degree of sinus opacification on preoperative CT imaging, were recruited from February 2021 to June 2021. Exclusion criteria consisted of patient refusal to participate in the study, American Society of Anesthesiology (ASA) grade greater than or equal to grade 3, body mass index (BMI) > 30, previous history of thromboembolic disease or allergy to TXA, long-term preoperative use of anticoagulants or antiplatelet drugs, or diagnosis of coagulation dysfunction. Informed written consent was obtained from all participants.

### Randomization and Blinding

Participants were randomized *via* a computer-generated random number list (by IBM SPSS Statistics 26.0) into one of two groups: the control group (Group C) or the TXA group (Group T). Each group included 30 patients. Group allocation for each participant was sealed in an envelope by one researcher who was blinded to the surgeons, patients, anesthesiologists, and recorders. The itinerate nurse, who was the only person aware of the allocation, opened these envelopes. In Group T, 15 mg/kg TXA (Beiruining, LUMMY, China) was dissolved in 100 ml normal saline. Thus, the drug administered in both groups was difficult for the researchers and surgeons to identify. The surgeries were performed by 2 surgeons with at least 15 years of ESS experience.

### Procedure

In Group T, patients received an intravenous drip of TXA at a dose of 15 mg/kg in 100 ml normal saline slowly over 30 mins. In Group C, patients received 100 ml of normal saline only. Next, all patients received a preload of 400 ml of sodium lactate ringer's injection irrigation before introduction. The protocol of general anesthesia was identical for all participants. Anesthesia was induced with midazolam 0.04 mg·kg^−1^, propofol 2 mg·kg^−1^, sufentanil 0.2 μg·kg^−1^, and cisatracurium 0.15 mg·kg^−1^ and was maintained with a continuous infusion of propofol and remifentanil. Volume-controlled ventilation at 5 to 8 ml/kg was applied *via* flexible laryngeal masks to keep the end-tidal carbon dioxide (Et-CO_2_) between 30 and 35 mmHg. The fraction of inspired oxygen (FiO_2_) was maintained at 70% in an oxygen–air mixture and all patients were placed at a 10-degree reverse Trendelenburg position before the surgery began. The bispectral index (BIS) (Covidien LLC, BIS VISTA, Mansfield, MA 02048 USA) was monitored and maintained between 40 and 60 by adjusting the infusion rate of propofol. Deliberate hypotension was applied. To maintain an intraoperative mean arterial pressure (MAP) between 65 and 75 mmHg and heart rate between 60 and 70 BPM, the injection speed of remifentanil was adjusted first. If this did not work, a bolus of 20 mg esmolol was used to decrease the heart rate, or 0.3 mg atropine was used to increase the heart rate. A bolus of 5 mg ephedrine or 0.2 mg nitroglycerin was administered when the intraoperative MAP was out of the aim range. Mucosal preparation was conducted with 10 cottonoid patties soaked in a 10 ml mixture of 1:10,000 epinephrine and 1% tetracaine and left *in situ* for 10 mins. Twenty other epinephrine-soaked patties were prepared for intraoperative hemostasis as required.

### Baseline Characteristics and Outcome Measures

The primary outcome variable was the Boezaart grading scale (BS) (Table 1) (9), which was assessed by a surgically trained researcher (who was blinded to the group allocation) *via* a display connected to the endoscope. Noninvasive blood pressure, heart rate (HR), and the BS score were recorded at 15-minute intervals during the operations. The secondary outcome variables were total blood loss (TBL), operation time (OT), and bleeding rate (BR). The TBL was calculated as the content in the suction container and sponges (subtracting the irrigation fluid), and the BR was calculated as the total blood loss divided by the surgical time. One milliliter of the venous blood sample was collected twice to measure whole blood coagulation and fibrinolysis by Sonoclot analysis (YKCA-1, Shijiyikang, Tianjin, China). The first sample was collected before the administration of TXA or normal saline as a baseline, and the second sample was obtained at the end of the operation. Six variables were recorded: the activated clotting time (ACT), the clot rate (CR), platelet function (PF), maximal clot signal (MCS), fibrin degradation time (FDT), and fibrin degradation rate (FDR). CR, ACT, and MCS correspond to coagulation functions depicting the rate of fibrin formation from fibrinogen, the time until thrombin generation and the beginning of fibrin formation (10), and the maximum strength of the clot, respectively. FDT and FDR are related to fibrosis function. Postoperative complications, including nausea, vomiting, anaphylaxis, visual impairment, seizure, venous thromboembolism (VTE), and postoperative intervention for excessive fresh bleeding in the first 24 h after the operation, were recorded.

**Table 1:** Boezaart score for the surgical field.

**Boezaart score**	**Description**
0	No bleeding
1	Slight bleeding—no suctioning is required
2	Slight bleeding—occasional suctioning is required. The surgical field is not threatened
3	Slight bleeding—frequent suctioning is required. Bleeding threatens the surgical field a few seconds after suction is removed
4	Moderate bleeding—frequent suctioning is required. Bleeding threatens the surgical field immediately after suction is removed
5	Severe bleeding—constant suctioning is required. Bleeding appears faster than can be removed by suction. The surgical field is severely threatened, and surgery not possible

### Statistical Methods

Sample size calculation was performed concerning the primary outcome variable, the BS score. Based on a previous study (11), 30 patients per group were necessary to detect a between-group difference of 10% with a type I error of 5% and a type II error of 20%. Data are summarized as the mean ± standard deviation (SD), median (interquartile range), or number (%), as appropriate. Quantitative data were compared by the unpaired t-test or Mann-Whitney U test as required between the two groups. Categorical data were analyzed using the chi-square test. Comparisons of coagulation and fibrinolysis function before and after the operation were performed using the Wilcoxon signed-rank test. Comparisons of serial changes in the BS score, HR, and MAP between the two groups were performed using a generalized estimating equation. Statistical analyses were performed using SPSS version 26.0 (IBM Corp, Armonk, NY). A *p* < 0.05 was considered statistically significant.

## Results

**Figure 1:**
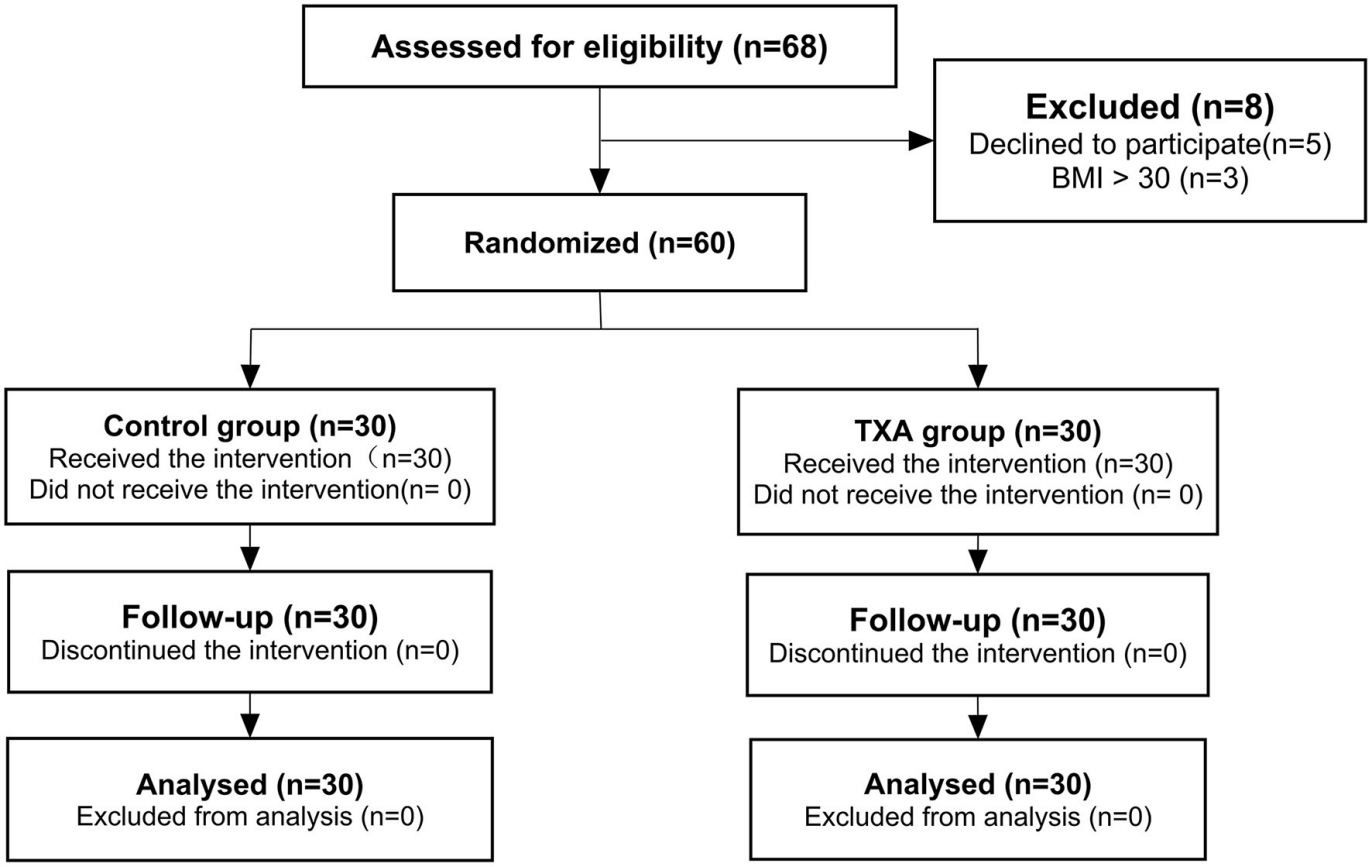
Consolidated Standards of Reporting Trials (CONSORT) flow chart showing the flow of patients through the trial. TXA, tranexamic acid.

A total of 68 cases were enrolled. Among them, 5 patients were excluded for declining to participate, and 3 were excluded for BMI greater than 30 kg/m^2^. The remaining 60 patients were randomly allocated to either the control group or the TXA group. With no patients lost to follow-up, the analysis was based on the data of 60 patients (Figure 1).

Baseline characteristics are presented in Table 2. No differences in demographics were noted between the two groups. The numbers of patients with nasal polyposis were similar between the two groups. The median Lund-Mackay scores were 20.0 (Group C) and 18.5 (Group T), and no difference was found.

**Table 2:** Baseline characteristics of the study patients^*^.

	**Group C**	**Group T**	***p***
Male/female	12/18	16/14	0.759^a^
Age, years	44.2 ± 12.6	44.1 ± 10.3	0.980^d^
BMI, kg/m^2^	24.3 ± 2.5	25.2 ± 3.0	0.224^d^
ASA 1/2	14/16	16/14	0.797^a^
Lund-Mackay scale score^e^	20.0 (14.8-22.0)	18.5 (15.8-22.0)	0.593^b^
Nasal polyposis, *n* (%)	25 (83.3)	22 (73.3)	0.532^a^
Eosinophils, %	6.09 ± 3.16	5.75 ± 3.11	0.683^d^
Preoperative oral steroids, *n* (%)	6 (20.0)	3 (10.0)	0.472^c^
Underlying diseases			
Hypertension, *n* (%)	2 (6.7)	7 (23.3)	0.145^c^
CHD, *n* (%)	3 (10.0)	0 (0.0)	0.237^c^
Diabetes, *n* (%)	2 (6.7)	2 (6.7)	1.0^c^
Asthma, *n* (%)	10 (33.3)	7 (23.3)	0.567^a^

* *Data are expressed as the mean ± SD, median (interquartile range), or number (%). Group C, control group; Group T, tranexamic acid group; BMI (Body mass index) = weight/(height)^2^; CHD, coronary heart disease*.

a *Pearson Chi-Square Test*.

b *Mann-Whitney U Test*.

c *Fisher's Exact Test*.

d *Unpaired T-test*.

e *The Lund-Mackay Scale assesses chronic rhinosinusitis severity by computed tomography*.

Table 3 lists the intraoperative characteristics. No differences in the mean MAP or mean HR were noted between the two groups. The application of ephedrine, esmolol, and nitroglycerin to maintain the intraoperative MAP between 65 and 75 mmHg and the intraoperative HR between 60 and 70 BPM was recorded, and no difference was found between the groups.

**Table 3:** Intraoperative characteristics^*^.

	**Group C**	**Group T**	***p* value**
MAP, mmHg	74.1 ± 6.0	72.6 ± 6.5	0.353^d^
HR, bpm	67.2 ± 9.6	69.8 ± 11.2	0.326^d^
Septoplasty, *n* (%)	11 (36.7)	11 (36.7)	1.0^a^
Ephedrine, *n* (%)	4 (13.3)	4 (13.3)	1.0^c^
BS (0–5)	2.27 (2.13–2.41)	2.02 (1.88–2.05)	0.011^b^
TBL, ml	374 (150–498)	300 (200–426)	0.668^b^
BR, ml/min	3.00 (1.50–4.22)	2.80 (1.97–3.52)	0.994^b^
OT, minutes	115.7 ± 32.4	105.5 ± 28.7	0.205^d^

* *Data are expressed as the mean ± SD, median (interquartile range), or number (%). Group C, control group; Group T, tranexamic acid group; BS, Boezaart scale score; TBL, total blood loss; BR, blood rate; OT, operation time; NRS, numeric rating scale; SD, standard deviation*.

a *Pearson Chi-Square test*.

b *Mann-Whitney U test*.

c *Fisher's Exact test*.

d *Unpaired T-test*.

**Figure 2:**
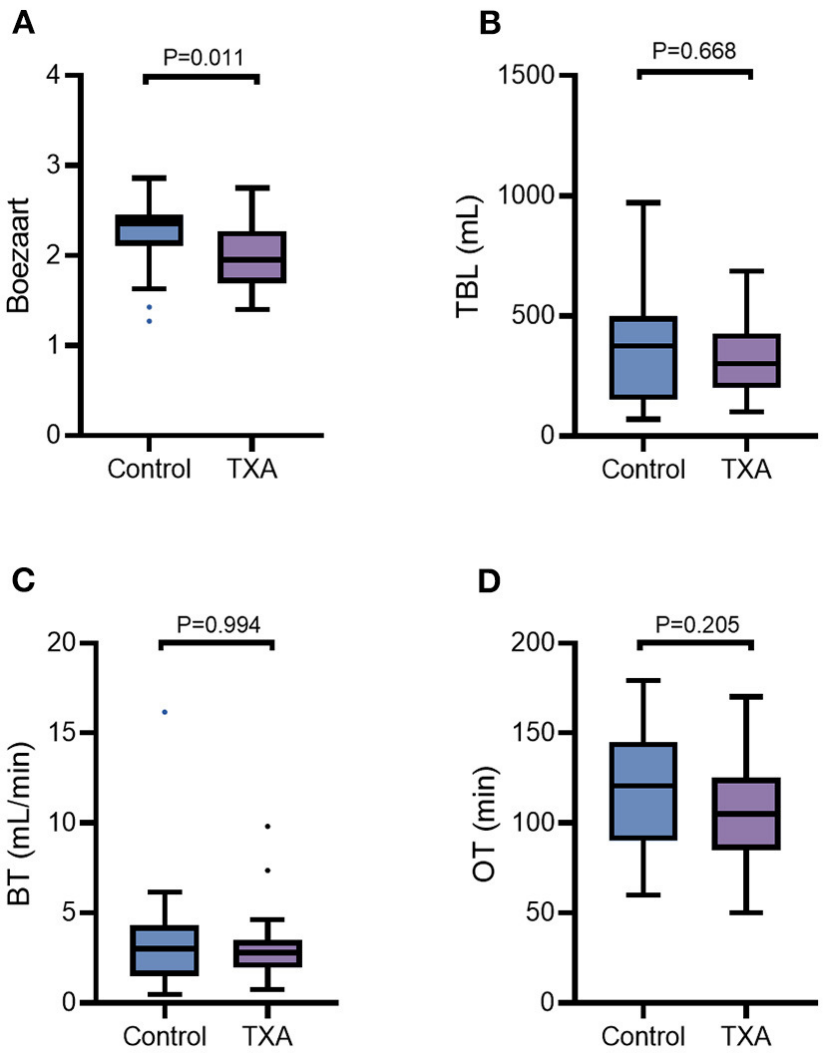
The Boezaart scores **(A)**, TBL **(B)**, BT **(C)**, and OT **(D)** were compared by the Mann-Whitney U test for comparisons between the two groups. The line in the middle represents the median value for each group, and the upper and lower bars represent the 25th and 75th percentiles, respectively. TBL, total blood loss; BT, bleeding rate; OT, operating time; TXA, tranexamic acid.

The bottom half of Table 3 and Figure 2 provide intraoperative visualization and bleeding data. The median and interquartile Boezaart scores were 2.27 (2.13–2.41) in Group C and 2.02 (1.88–2.05) in Group T, respectively, with a significant difference noted between the two groups (*p* = 0.011). However, the difference in TBL was not statistically significant between Group C and Group T [374 (150–498) ml vs. 300 (200–426) ml, P = 0.668]. Similarly, there were no differences in BR [3.00 (1.50–4.22) ml/min vs. 2.80 (1.97–3.52) mL/min, *p* = 0.994] and OT (115.7 ± 32.4 mins vs. 105.5 ± 28.7 mins, *p* = 0.205) between Group C and Group T.

The incidences of postoperative complications in the 60 patients are shown in Table 4, and no significant difference was noted between the two groups. In the first 24 h after the operation, there were no severe complications, such as anaphylaxis, visual impairment, seizures, venous thromboembolism (VTE), or acute dysfunction of vital viscera. No patients exhibited excessive fresh bleeding that required medical intervention in the first 24 h after the operation.

**Table 4:** Postoperative complications^*^.

	**Group C**	**Group T**	***p*-value**
Nausea, *n* (%)	3 (10)	4 (13.3)	1.0
Vomiting, *n* (%)	2 (6.7)	3 (10.0)	1.0
Headache, *n* (%)	3 (10)	4 (13.3)	1.0
Dizziness, *n* (%)	2 (6.7)	3 (10)	1.0

* *Data are expressed as the number (%). Group C, Control group; Group T, tranexamic acid group*.

**Figure 3:**
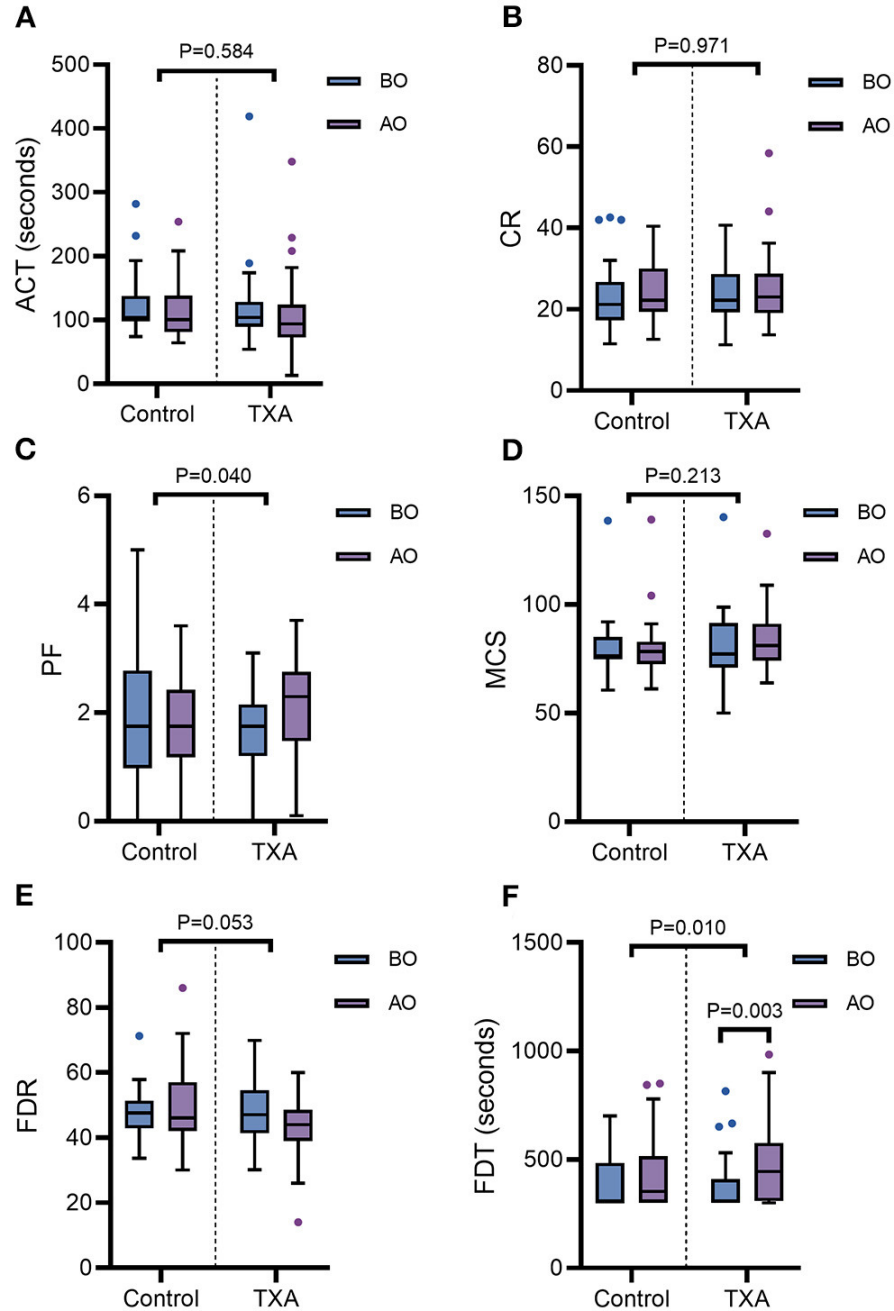
The ACT-D **(A)**, CR-D **(B)**, PF-D **(C)**, MCS-D **(D)**, FDR-D **(E)**, and FDT-D **(F)** were compared by the Mann-Whitney U test for comparisons between the two groups. The line in the middle indicates the median value for each group, and the upper and lower bars represent the 25th and 75th percentiles, respectively. TXA, tranexamic acid; ACT, activated clotting time; CR, clot rate; PF, platelet function; MCS, maximal clot signal; FDT, fibrin degradation time; FDR, fibrin degradation rate; BO, before the operation; AO, after the operation.

The results of the Sonoclot analysis are shown in Figure 3. Compared with the baseline, changes in CR, ACT, PF, MCS, and FDR at T2 were not significant in either of the groups (*p* > 0.05). However, in Group T, FDT had a significant increase at T2 compared with T1 [445 (309–576) vs. 308 (300–410), *p* = 0.003]. There was also an increase in PF at T2 [2.30 (1.48, 2.75)] compared with T1 [1.75 (1.20, 2.15)] in Group T, but this increase was not statistically significant (*p* = 0.053). In Group C, no significant changes were found in FDT during the operation. The changes in CR, ACT, MCS, and FDR from T1 to T2 were not significant between the two groups (*p* > 0.05). The increase in PF and FDT from T1 to T2 in Group C was significantly lower than that in Group T (*p* = 0.040 for PF; *p* = 0.010 for FDT).

**Figure 4:**
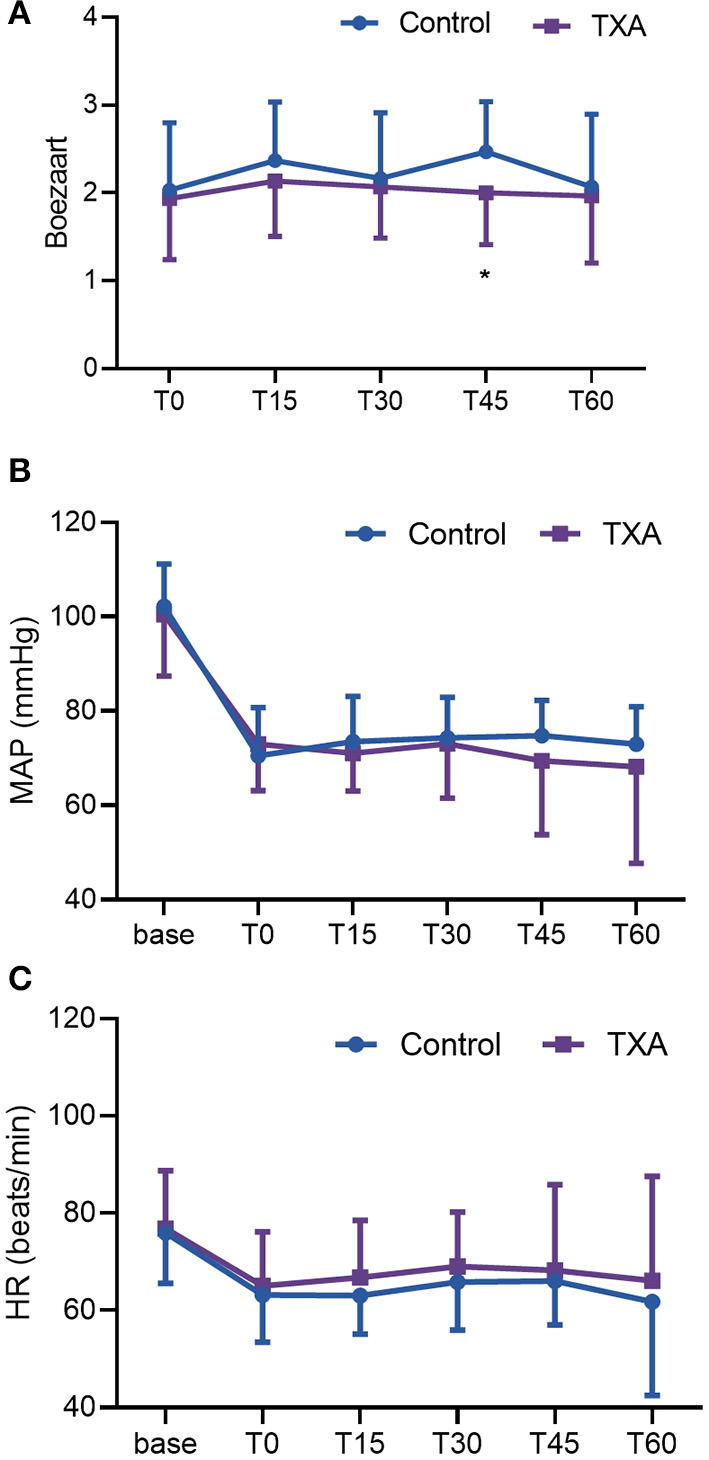
**(A)** Boezaart scores in the first 60 mins. **(B)** MAP in the first 60 mins. **(C)** HR in the first 60 mins. The dots represent the means of each group at the following times: before anesthesia (base); the beginning of the operation (T0); and 15 mins (T15), 30 mins (T30), 45 mins (T45), and 60 mins (T60) into the operation. One-sided bars represent the standard deviation. TXA, tranexamic acid; HR, heart rate; MAP, mean arterial pressure.

Figure 4 shows the BS score, MAP, and HR data during the first 60 mins of the operations. There were no differences in MAP and HR between the two groups, but a significant difference in the BS score at T45 (45 mins after the beginning of surgery) was found between the two groups.

## Discussion

This randomized, controlled, double-blinded study verified the potency of intravenous TXA in improving the intraoperative visualization of ESS for high-grade chronic rhinosinusitis without increasing complications.

Several prospective studies have evaluated the effect of intravenous TXA on the surgical visualization of ESS. However, the results have been inconsistent. A systematic review (6) found that TXA improved the surgical field and reduced blood loss during nasal surgery. The meta-analysis performed by Kim et al. (5) found that TXA reduced blood loss and operative time in endoscopic sinus surgery based on a small number of enrolled studies. Langille et al. (7) applied a bolus TXA of 15 mg/kg with a continuous TXA infusion at 1 mg/kg/h and did not find decreased efficiency in the surgical field or intraoperative blood loss. The difference is that the median Lund-Mackay scores (10–11) of the patients in their study were lower than those in this trial (18.5–20.0). A previous study (3) showed that the Lund-Mackay score can predict intraoperative bleeding for endoscopic ethmoidectomy, and specific measures (such as preoperative corticosteroids) are suggested for patients with high Lund-Mackay scores. In this trial, only patients with Lund-Mackay scale scores greater than 12 were enrolled, characterizing a group of the population that had advanced paranasal sinus disease and severe mucosal inflammation. In a randomized controlled study (12), Brunner found that total intravenous anesthesia (TIVA) can result in better surgical visualization than inhaled anesthesia for high-grade sinus disease. We applied TIVA as a routine technique in this study and still found that TXA exerted a beneficial effect on the surgical visualization of ESS for high-grade chronic rhinosinusitis.

Fibrinolysis is initiated to limit clot formation following tissue and vascular injury by the activation of plasminogen. In addition to lysing fibrin, plasmin can mediate the activation of multiple hemostatic and inflammatory components. By cleaving glycoprotein Ib and IIb/IIIa receptors on platelets, fibrinolysis can reduce platelet adhesion and aggregation, thus contributing to coagulopathy (13). As a synthetic antifibrinolytic agent, TXA can block the lysine-binding sites on plasminogen and inhibit its affinity to fibrin. In this study, the relatively increased PF and prolonged FDT with the use of TXA may account for the better surgical visualization in the TXA group.

TXA is beneficial to cardiac and major orthopedic surgeries, and despite its mechanism of coagulation and fibrinolysis, the risk of venous thromboembolism has not been detected to increase (13). Similarly, with a bolus dose of 15 mg/kg in this study, the incidence of complications in the TXA group was not increased compared to that in the control group. A total dose of 1.0 g for adults is suggested by Ker et al. to reduce surgical bleeding, and complications, such as seizures, may be related to high-dose (≥100 mg/kg) administration (14).

In this study, despite the better surgical visualization in the TXA group, the reduction in blood loss and OT by TXA administration was not significant, which differs from the findings in a previous study (5). This difference might be related to the severity of the disease. With nasal polyposis and swelling of the sinus mucosa, the interspace for operation can be especially narrow, and minimal bleeding can quickly obscure the surgical field (1) and increase the Boezaart scores of surgical visualization. It can be inferred that a small reduction in bleeding might improve the surgical field. Another factor is that the surgeons participating in this study were proficient (with at least 10 years of experience in endoscopic sinus surgeries) and may have been more expert in dealing with obscured visualization than a beginner. In addition, the combined use of controlled hypotension and TIVA, which is commonly used to reduce bleeding clinically, probably reduced the effect of TXA.

## Limitations

There are some limitations to this study. First, the rating system of surgical visualization is subjective with limited reliability. In this study, one researcher blinded to the group allocation assessed the BS scores of the two groups to make them comparable. Second, the enrolled patients were relatively healthy, which may restrict the clinical application of the conclusion extending to the whole population. Third, the volume of postoperative bleeding, which might indicate the postoperative effect of TXA, was not recorded. Finally, the number of patients in the study was small and may not have been sufficient to observe any serious complications with the use of TXA. Therefore, to assess the safety of TXA for ESS, more observations and studies should be performed.

## Conclusions

A 15 mg/kg bolus of intravenous TXA before surgery can improve the surgical visualization of ESS for high-grade chronic rhinosinusitis without causing significant adverse effects. Intravenous TXA may be beneficial in ESS for high-grade chronic rhinosinusitis.

## Data Availability Statement

The raw data supporting the conclusions of this article will be made available by the authors, without undue reservation.

## Ethics Statement

The studies involving human participants were reviewed and approved by the Ethics Committee of Beijing Tongren Hospital (TRECKY2020-065). The patients/participants provided their written informed consent to participate in this study.

## Author Contributions

GW: work design and made important changes to the paper. HG, HL, YL, YW, and CW: data collection. WY: draft papers. LZ: approved the final version of the paper to be published. All authors contributed to the article and approved the submitted version.

## Funding

This study was supported by Beijing Hospitals Authority Clinical Medicine Development of Special Funding Support (ZYLX202103). The funders had no role in the study design, data collection and analysis, decision to publish, or preparation of the manuscript.

## Conflict of Interest

The authors declare that the research was conducted in the absence of any commercial or financial relationships that could be construed as a potential conflict of interest.

## Publisher's Note

All claims expressed in this article are solely those of the authors and do not necessarily represent those of their affiliated organizations, or those of the publisher, the editors and the reviewers. Any product that may be evaluated in this article, or claim that may be made by its manufacturer, is not guaranteed or endorsed by the publisher.
